# Differentiated Service Delivery Models for HIV Treatment in Malawi, South Africa, and Zambia: A Landscape Analysis

**DOI:** 10.9745/GHSP-D-20-00532

**Published:** 2021-06-30

**Authors:** Amy Huber, Sophie Pascoe, Brooke Nichols, Lawrence Long, Salome Kuchukhidze, Bevis Phiri, Timothy Tchereni, Sydney Rosen

**Affiliations:** a Health Economics and Epidemiology Research Office, Wits Health Consortium, Department of Internal Medicine, School of Clinical Medicine, Faculty of Health Sciences, University of the Witwatersrand Johannesburg, South Africa.; b Department of Global Health, Boston University School of Public Health, Boston, MA, USA.; c Clinton Health Access Initiative, Lusaka, Zambia.; d Clinton Health Access Initiative, Lilongwe, Malawi.

## Abstract

**Introduction:**

Many countries in Africa are scaling up differentiated service delivery (DSD) models for HIV treatment, but most existing data systems do not describe the models in use. We surveyed organizations that were supporting DSD models in 2019 in Malawi, South Africa, and Zambia to describe the diversity of DSD models being implemented at that time.

**Methods:**

We interviewed DSD model implementing organizations for descriptive information about each of the organization's models of care. We described the key characteristics of each model, including population of patients served, location of service delivery, frequency of interactions with patients, duration of dispensing, and cadre(s) of provider involved. To facilitate analysis, we refer to 1 organization supporting 1 model of care as an “organization-model.”

**Results:**

The 34 respondents (8 in Malawi, 16 in South Africa, 10 in Zambia) interviewed described a total of 110 organization-models, which included 19 facility-based individual models, 21 out-of-facility-based individual models, 14 health care worker-led groups, and 3 client-led groups; jointly, these encompassed 12 specific service delivery strategies, such as multimonth dispensing, adherence clubs, home delivery, and changes to facility hours. Over two-thirds (n=78) of the organization-models were limited to clinically stable patients. Almost all organization-models (n=96) continued to provide clinical care at established health care facilities; medication pickup took place at facilities, external pickup points, and adherence clubs. Required numbers of provider interactions per year varied widely, from 2 to 12. Dispensing intervals were typically 3 or 6 months in Malawi and Zambia and 2 months in South Africa. Individual models relied more on clinical staff, while group models made greater use of lay personnel.

**Conclusions:**

As of 2019, there was a large variety of differentiated service models being offered for HIV treatment in Malawi, South Africa, and Zambia, serving diverse patient populations.

## Abstract (precis)

Observing the diversity of differentiated service delivery models for HIV treatment in use in sub-Saharan Africa can help policy makers and program planners to improve decision making for treatment delivery in the future. This effort can inform decisions about how to optimize the distribution of models across facilities and regions and how to plan for budget and resource allocation.

## Teaser

**Key Findings:**

- Differentiated service delivery (DSD) models for HIV treatment in Malawi, South Africa, and Zambia can be grouped into 12 service delivery strategies that vary by population served, medication dispensing duration, location of medication delivery, frequency of health care system interactions, and other characteristics.
- As of 2019, most DSD models in Malawi, South Africa, and Zambia remained limited to clinically stable, adult patients and continue to depend on established facilities for clinical care; individual models relied more on clinical staff, while group models made greater use of lay personnel.
- DSD models required anywhere from 2 to 12 health care system interactions per year, imposing very different burdens on patients and clinics.

**Key Implications:**

- Policy makers should recognize that each DSD model is designed differently and requires different resources for implementation; the details of how a specific model operates are important to understanding the optimal model mix for future scale-up.
- Existing routine data systems do not capture patients' participation in specific DSD models, making it difficult to assess model coverage or performance. Improving electronic medical record systems so that they reflect actual service delivery is a high priority.

## INTRODUCTION

In 2019, approximately 17.8 million people were receiving antiretroviral therapy (ART) for HIV in sub-Saharan Africa.^1^ Achieving global targets for HIV treatment, known as “95-95-95,”^2^ would require approximately another 5 million patients to be added to the national HIV treatment programs. Meanwhile, donor spending in low- and middle-income countries is declining, which has led countries, implementers, and funders to seek avenues of greater efficiency in service delivery.^3^^,^^4^

One response to this challenge is the development of “differentiated service delivery models” (DSD models) for HIV treatment.^5^ DSD models typically reduce clinic visits and/or move services out of the clinic and may also alter the provider cadre and package of services provided. These models have multiple aims. They seek to make treatment more patient-centric by lessening the burden of frequent clinic visits; reduce costs to both the health care system and to patients; and sustain or improve clinical treatment outcomes.^6^ DSD models are intended as an alternative to traditional or conventional care, which has typically been delivered at fixed-site clinics and requires at least 4 patient visits to the clinic per year, if not more. DSD models were originally targeted to adults in the general population who are “stable” on treatment and who comprise the largest population of patients. Models for other populations such as children and those with detectable viral loads have also been developed.

DSD models seek to lessen patients' burden of frequent clinic visits, reduce patient and health care system costs, and sustain or improve clinical treatment outcomes.

Although scale-up of DSD models is well underway in many sub-Saharan African countries, existing data systems have not yet caught up with the diversity of approaches to HIV treatment delivery. Documentation of care delivered through DSD models is either not captured or is poorly captured in existing electronic medical record systems, and even paper-based patient files and site-level registers may not record, for example, whether a patient received a medication refill at the site, at a community pick-up point, or at home.^7^^,^^8^ In most countries, moreover, a wider range of different models are being implemented than may be reflected in national HIV treatment guidelines, with both ministries of health and nongovernmental partners designing and piloting approaches that vary from those in the guidelines. Some of these models are described in the published^9^^–^^12^ or unpublished^13^^,^^14^ literature or in funder or government databases, but most countries lack a comprehensive inventory of what is being tried. Policy makers thus lack familiarity with the range of DSD experimentation underway, let alone being well-informed about the strengths and weaknesses of different models or their implications for the health care system.

Policy makers may not be fully familiar with the range of DSD model experimentation underway and may not be well-informed about the strengths and weaknesses of different models or their implications for the health care system.

In 2019, we undertook a series of interviews with DSD model implementing organizations in Malawi, South Africa, and Zambia to describe the current landscape of DSD model implementation in 3 high HIV prevalence countries in southern Africa, each representing a different income level (lower, upper-middle, and middle income, respectively). Interviews were conducted with as many nongovernmental and governmental implementing organizations and agencies as could be identified. Here, we present the information obtained through these interviews on the types and characteristics of DSD models underway. Our goal is to illustrate the diversity of models in use, identifying similarities and differences, in the hope that a knowledge of the breadth of models being tried will help policy makers and program planners to improve decision making for DSD models in the future.

## METHODS

We conducted cross-sectional structured interviews with DSD model implementing organizations in Malawi, South Africa, and Zambia. Each interview elicited the title and implementation start date for each differentiated model of care implemented by the organization and then collected descriptive information about the model, including population eligible, location and frequency of service delivery, provider cadre, and scale. Respondents were also asked for information on data and documentation, existing evaluations, and future plans for DSD projects.

### Identification of Respondents

Our goal was to survey all the organizations that were either implementing or supporting implementation of DSD models in the study countries at the time of the survey (2019), whether for purposes of routine care, demonstration or pilot projects, or research studies. Potential survey respondents included government health agencies, implementing partners of national governments and of the U.S. President's Emergency Plan for AIDS Relief (PEPFAR) and other donors, and other nongovernmental organizations. We first compiled a comprehensive list of potential respondents. We started with the study team's own knowledge of each country's HIV program and then supplemented this with recommendations from government DSD technical working groups and funding organization representatives. We then reviewed each country's list with relevant national government representatives. Once the inventory of potential respondents was complete, each organization was invited to participate in the survey. Over the course of the survey, we also asked respondents to review and add to the list of potential participants from their own countries.

### Data Collection

Data were collected using a semistructured questionnaire administered by the study team in a face-to-face or electronic meeting with a representative of each participating organization. Interviews were audio-recorded and data entered into the project database after each interview was completed. Questions were limited to factual information about the DSD models being implemented. The interview instrument is included in Supplement 1. After all data had been entered, a report of the responses was sent to each respondent for verification, correction of errors, and provision of specific information that was not available at the time of the interview, such as the precise numbers of patients participating in each model.

### Data Analysis

As this was a descriptive analysis, we aimed to group models within each country by their major characteristics, so that an overall profile of the models in use in each country could be developed. We first categorized the models using the taxonomy by Grimsrud et al.,^15^ which is widely used within the DSD model literature. This taxonomy sorts models of care into 4 categories: facility-based individual models (FBIM), such as fast-track clinic visits; out-of-facility-based individual models (OFBIM), such as decentralized medication delivery; health care worker-led groups (HCWLG), such as adherence clubs; and client-led groups, such as community adherence groups or CAGs.

Although this 4-category taxonomy is widely used, it also results in combining very diverse models, such as home delivery of ARVs and community-based clinical care, into the same category. We therefore also created a set of “strategies” that further refines the categories, grouping more similar models together. As we defined these strategies empirically, based on the interview responses, we regard them as results of the survey and present them in the results section.

We then used interview data to describe the key characteristics of each model, adapted from the well-known domains proposed by Duncombe et al.^5^: population of patients served, location of service delivery, frequency of interactions with patients, duration of dispensing, and cadre(s) of provider involved (Supplement 2). For each characteristic in the results section, we start by describing traditional or conventional care, to provide a comparison with DSD model characteristics. We then report frequencies of each characteristic in each domain.

In reporting aggregate results, it is important to note that each organization-model combination was counted once, regardless of how many clinics offered the model, how many patients were enrolled, or whether other partners described the same model. For example, in this analysis a model being piloted at 2 clinics, with just a few dozen patients enrolled, and a model that had already been scaled up nationally and covered hundreds of thousands of patients were counted equally. Similarly, if 2 respondent organizations each responded that they were implementing the same model, this model was counted twice. We refer to each combination of 1 model described by 1 respondent as an “organization-model.”

Although we did ask survey respondents to report the numbers of sites or facilities implementing each model and of patients participating, in most cases we were unable to obtain complete or accurate data for these. Where such numbers were available, it was generally not possible to confirm that no patients were double-counted in other implementer reports or that no individual patients were enrolled in more than 1 model. We therefore do not include information here on the scale of model implementation or coverage.

## RESULTS

### Interviews Conducted and Models Reported by Respondents

We identified 36 potential respondents in the 3 countries and completed interviews with 34 of them. The remaining 2, both in South Africa, declined to participate in the survey. Interviews were conducted between March 2019 and March 2020, with data verified by respondents between November 2019 and March 2020. We surveyed a total of 8 organizations in Malawi, 16 in South Africa, and 10 in Zambia.

The 34 respondents interviewed reported on a total of 110 organization-models, where an organization-model represents 1 organization supporting 1 model, as shown in the upper half of Table 1. Some models are specified in each country's HIV treatment guidelines, to be scaled up nationally; others are bespoke models originating from nongovernmental organizations. Countries differed in their most commonly reported category of DSD model: more organizations in Malawi described facility-based individual models and more in Zambia described out-of-facility-based individual models, while no organizations at all in South Africa reported supporting client-led group models.

**TABLE 1.** Number of Organization-Models^a^ for Differentiated Service Delivery of HIV Treatment Described by Respondents, by Country, Category, and Strategy

Model Category	Malawi	South Africa	Zambia	Total
Total number of organization-models described	26	43	41	110
**Number of organization-models per country and category**				
Facility-based individual model	13	6	13	32
Out-of-facility-based individual model	5	22	16	43
Health care worker-led group	7	15	4	26
Client-led group	1	0	8	9
**Numbers of organization-models per country and strategy**				
Adherence clubs	0	11	3	14
Community adherence groups	1	0	8	7
Community outreach	3	3	4	11
External pickup points	2	17	6	26
Extra clinic hours	1	1	2	4
Family models	2	1	1	4
Fast track services	2	1	5	8
Home delivery	0	1	3	4
Multimonth dispensing	3	1	3	7
Nonstable patient models	6	4	1	11
Key population models	1	1	2	4
Youth models	5	2	3	10

a An organization-model is 1 organization supporting 1 model of care.

We also grouped the 110 organization-models into 12 strategies, as described in Table 2 and listed alphabetically in the lower half of Table 1. The 12 strategies do not map directly onto the 4 categories; each taxonomy provides different information.

**TABLE 2.** Twelve Strategies for Differentiated Service Delivery Model for HIV Treatment in Malawi, South Africa, and Zambia, as Reported by Interview Respondents

Adherence clubs	Any group model that is led by a health care worker (professional or lay)
Community adherence groups	Any group model in which a patient picks up medications for other group members (typically abbreviated CAG)
Community outreach	A variety of models that bring both clinical care and medications into the community, such as nurse-led outreach
External pickup points	Any model that delivers antiretroviral medications to pickup points outside clinic facilities, such as lockers, community pharmacies, decentralized pickup points, etc.
Extra clinic hours	Any model that adds additional hours to a facility's operations to facilitate access, such as on evenings or weekends
Family models	Any model designed to serve multiple and/or specific members of a family at once (e.g., pediatric clinic or family clinic)
Fast track services	Any model that creates a separate queue, kiosk, or procedure at a facility to speed up service delivery for stable patients
Home delivery	Any model that delivers antiretroviral medications to patients' homes (e.g., by a community health worker or a bicycle courier)
Multimonth dispensing	Any model in which the primary goal is to dispense medications for a longer duration than is done under standard care (usually 6 months)
Nonstable patient models	Models for patients who do not meet definitions of clinical stability, such as high viral load clinics and advanced disease clinics
Key population models	Models for a key population such as men who have sex with men or female sex worker
Youth models	Any model specifically for youth/teens/adolescents (e.g., teen clubs in Malawi and the scholar model in Zambia)

There are several provisos to the model taxonomies in Table 1. First, while we used both the names given to the models by the implementing organizations and interview respondents' descriptions of the models to allocate each organization-model to a category and approach, in some cases we were uncertain and had to choose what appeared to be the closest fit, given what we knew. Second, not all of the models listed in Table 1 are mutually exclusive. For example, 6-month dispensing can be implemented within many other models of care. Third, many models also provide some services that are not strictly consistent with their model category or approach. Out-of-facility-based individual models, for example, may provide some services at facilities, while facility-based models may include home visits for patients who miss appointments; both group model categories likely include some individual services. Similarly, community outreach strategies may incorporate external medication pickup points, along with community-based clinical care. Finally, a model that is considered “differentiated” in 1 country—like 3-month dispensing in South Africa—and is thus included in Table 1 may be regarded as standard of care in another, like Malawi, for which it is not mentioned in Table 1.

### Populations Served

As anticipated, most of the models described in the survey served adults in the general population who were stable on treatment. Definitions of stability varied by country and model, but most included a minimum of 12 months on ART and evidence of viral suppression. A number of other models were designed for people with advanced HIV disease, an unsuppressed viral load, or newly initiated on ART. Models also existed for different age groups (children, adolescents) and vulnerability groups (men who have sex with men (MSM), female sex workers (FSW), pregnant women). We note that the survey was completed before the adoption of coronavirus disease (COVID-19) related changes to eligibility criteria, which may or may not be permanent. In Table 3, the number of organization-models for each population group is reported by country.

**TABLE 3.** Populations Served by Current Differentiated Service Delivery Models for HIV Treatment and Criteria for Defining Stability, by Country

Population or Requirements	Malawi, No.	South Africa, No.	Zambia, No.	Total, No.
**Population eligible**				
Number of organization-models	26	43	41	110
All patients (no restrictions by disease status or age)	2	1	0	3
Stable and not stable patients				
Adults and adolescents/youth	0	2	0	2
Adolescents/youth (age restrictions vary)	5	2	0	7
Children (age restrictions vary)	0	0	1	1
Total stable and not stable patients	5	4	1	10
Stable patients only				
All ages	0	0	10	10
Adults	10	31	14	55
Adults and adolescents/youth	0	0	9	9
Adolescents/youth (age restrictions vary)	0	0	3	3
Children (age restrictions vary)	0	1	0	1
Total stable patients only	10	32	36	78
Advanced disease/not stable patients only				
All ages	4	2	1	7
Adults	2	2	0	4
Children (age restrictions vary)	1	0	0	1
Total advanced disease/not stable patients	7	4	1	12
Pregnant/postpartum women only (any disease status)	1	1	0	2
MSM/FSW (any disease status)	1	1	3	5
**Requirements to be regarded as stable**				
Number of organization-models	10	32	36	78
ART ≥ 6 months and 1 suppressed viral load	5	4	9	18
ART ≥ 12 months and 1 suppressed viral load	1	0	23	24
ART ≥ 12 months and 2 suppressed viral loads	0	26	0	26
Not specified	4	2	4	10

Abbreviations: ART, antiretroviral therapy; FSW, female sex worker; MSM, men who have sex with men.

Half (n=55, 50%) of the organization-models were limited to stable adults, and more than two-thirds (n=78, 71%) to stable patients overall. About a quarter (n=25, 23%) either accepted advanced disease or unsuppressed patients along with stable patients or were explicitly designed for those with advanced disease or viral failure. A small handful targeted special populations (n=7, 6%).

As mentioned, where “stability” was a criterion for DSD model enrollment, definitions varied. In Table 3, we also summarize the criteria used for the 78 organization-models limited to stable patients. Of the 70 organization-models for which stability criteria were specified, more than two-thirds (n=50, 71%) required that patients have spent at least a full year on ART before DSD model eligibility, and all required at least 1 suppressed viral load measurement.

### Location

Locations of models reported by survey respondents are shown in Table 4. As mentioned above, one of the main criteria for differentiating HIV treatment delivery from traditional, clinic-based care is the location of service. Before differentiation, nearly all care and medication dispensing took place at fixed-site clinics, with occasional community outreach efforts to trace defaulters or provide treatment education or adherence support. DSD models offer services in a wide range of locations, from fixed-site clinics to private pharmacies and community meeting spaces to patients' homes. For models that provide most services off-site, patients typically remain the responsibility of a fixed-site clinic, which supervises the delivery of care through the alternative model and maintains patient records.

**TABLE 4.** Number of Organization-Models for Differentiated Service Delivery of HIV Treatment by Location of Service Delivery and Country

Location of Service Delivery	Number of Organization-Models			
	Malawi N=26	South Africa N=43	Zambia N=41	Total N=110
Facility for clinical care with medication pickup at				
Facility	19	6	16	41
External pickup point	4	18	15	37
Adherence club	0	13	1	14
Home	0	1	3	4
Total (facility location for clinical care)	23	38	35	96
External location for clinical care				
Clinical care and medications in community	3	3	5	11
Clinical care and medications at adherence club	0	2	0	2
Clinical care and medications at home	0	0	1	1
Total (external location for clinical care)	3	5	6	14

Almost all of the organization-models (n=96, 87%) continued to provide clinical care at established health care facilities, though each country had a few organization-models that delivered clinical care outside the facility. Medication pickup locations varied by country. Facility-based pickup was most common among organization-models in Malawi; external pickup points and pickup at adherence clubs, frequently located at the facility rather than in the community, were widely used in South Africa; and medication pickup at facilities and at external pickup points were both common in Zambia.

### Frequency of Interactions With Health Care System

In addition to location of service delivery, the number of times per year that a patient must interact with the health care system—either an established clinic or an off-site location—is a critical differentiator of the alternative models of care that have been developed. During the first decade of large-scale, public-sector ART programs in Africa, patients typically collected their medications once a month, with clinical monitoring conducted 4 to 12 times per year. Dispensing intervals expanded gradually, from a maximum of 1 month at a time to up to 3 months in some countries, but frequent clinic visits remained the norm.

In an effort to reduce the burden of treatment on patients and clinics, where time and space should be freed up by requiring fewer ART visits per year, DSD models generally try to keep stable patients out of the clinic. This may be done by reducing the absolute number of clinic visits per year and/or replacing some or all traditional or “full” clinic visits with briefer visits for medication pickup only, such as fast-track visits, or off-site interactions, such as adherence club participation or medication access at an external pickup point. Table 5 summarizes the number of clinic and DSD model interactions required per year, with “clinic visit” referring to a full or traditional visit and “DSD interaction” including short clinic visits or medication refill/pick-up visits that were designed for a DSD model. Models that required evidence of viral suppression for eligibility are reported separately from those that did not. While the total number of clinic visits and DSD interactions required per year varied widely by model, most organization-models continued to expect patients to interact with health care providers, either at or outside the facility, at least 4 times per year.

**TABLE 5.** Frequency of Health Care System Interactions per Year, by Country, Model Category, and Viral Suppression Eligibility Criterion for Differentiated Service Delivery Models for HIV Treatment

Frequency of Interactions Required per Year	Malawi			South Africa			Zambia		
	Organization-models, No.	Full Clinic Visits,^a^ Median (Range)	Other Interactions,^b^ Median (Range)	Organization-models, No.	Full Clinic Visits,^a^ Median (Range)	Other Interactions,^b^ Median (Range)	Organization-models, No.	Full Clinic Visits,^a^ Median (Range)	Other Interactions,^b^ Median (Range)
**Models explicitly for stable patients**									
**Facility-based individual models**									
Every 2 months (6/year)				1	4	0			
Every 3 months (4/year)	2	2 (2–2)	2 (2–2)	1	2	4	8	2 (2–4)	2 (0–2)
Every 4 months (3/year)	3	2 (2–2)	1 (1–1)						
Every 6 months (2/year)							3	2 (2–2)	0 (0–0)
**Out-of-facility-based individual models**									
Every 2 months (6/year)				18	2 (2–2)	4 (4–4)			
Every 3 months (4/year)	3	1 (0–2)	4 (2–4)	1	0	5	13	2 (0–2)	2 (2–4)
**Health care worker-led group models**									
Every 2 months (6/year)				10	2 (2–2)	4 (4–4)			
Every 3 months (4/year)	1	2	2				4	2 (2–4)	2 (0–2)
Every 6 months (2/year)				1	0	2			
**Client-led group models**									
Every 1 month (12/year)							1	2	10
Every 3 months (4/year)	1	2	2				6	2 (2–2)	2 (2–2)
Every 6 months (2/year)							1	1	1
**Models not requiring stability^c^**									
**Facility-based individual models**									
Every 1 month (12/year)	6	0 (0–0)	12 (12–12)	2	0 (0–0)	12 (12–12)	2	0 (0–0)	12 (12–12)
Every 2 months (6/year)				2	0 (0-0)	6 (6–6)			
Every 3 months (4/year)	1	0	4						
Visits aligned to PMTCT schedule	1	5	3						
**Out-of-facility-based individual models**									
Every 2 months (6/year)				3	0 (0–0)	6 (6–6)			
Every 3 months (4/year)	2	0 (0–0)	4 (4–4)				3	2 (2–2)	2 (2–2)
**Health care worker-led group models**									
Every 1 month (12/year)	2	0 (0–0)	12 (12–12)	3	0 (0–2)	12 (10–12)			
Every 2 months (6/year)	2	0 (0–0)	6 (6–6)						
Visits aligned to child vaccine schedule				1	0	8			
**Not specified**	2								

a A full clinic visit is a conventional facility visit that provides the services that were typical of a predifferentiation visit, generally including a consultation with a clinician, counseling, laboratory tests if scheduled, and a medication refill.

b Other interactions include any interaction with a provider that has been tailored for the population served, including off-site medication pickups, adherence club participation, or a clinical consultation adjusted for the population in question, including specialist consultations for certain types of patients (i.e., all interactions except conventional, undifferentiated full clinic visits).

c Includes models for known unsuppressed patients, high viral load clinics, pediatric clinics, and models that accept both stable and nonstable patients.

### Dispensing Intervals

Interaction frequency, as presented in Table 5, appears to be determined in part, but not solely, by duration of dispensing; if patients receive a 3-month supply of ARVs at a time, interactions must take place at least quarterly. However, many models are designed to interact with patients more frequently than once per quarter. As previously mentioned, early in national ART programs, patients received a maximum of 1 month of medications at a time, and refills were dispensed only by fixed-site clinics. Because a medication refill visit often takes a full day due to long waiting times—even if a clinical consultation is not required—a promising way to improve treatment delivery is to dispense more months at a time. “Multimonth dispensing” (i.e., at least 3 months of medication dispensed) is now the norm in Zambia and Malawi and under consideration in South Africa, but the number of months allowed in our survey varied from 2 to 6. Table 6 summarizes dispensing intervals expected for DSD models in each country.

**TABLE 6.** Months of Antiretrovirals Dispensed in Differentiated Service Delivery Models for HIV Treatment

Average Months of Antiretrovirals Dispensed in Model	Number of Organization-Models			
	Malawi (N=26)	South Africa (N=43)	Zambia (N=21)	Total (N=110)
1 month	7	5	3	15
2 months	2	34	0	36
3 months	7	1	12	20
1 or 3 months (patients typically start with 1 month, then move to 3 months)	2	2	2	6
6 months	3	1	7	11
3 or 6 months (typically previously dispensed 3 months but transitioning to 6 months in line with national policy)	3	0	17	20
Not reported	2	0	0	2

Dispensing intervals varied in Malawi, with some organization-models dispensing only 1 month at a time and others offering 6 months per pickup. This variation in dispensing is related to patient population served, with special populations and those with an unsuppressed viral load generally receiving shorter intervals and stable patients generally receiving 3 or 6 months. In South Africa, 2-month dispensing remained the norm, with only 1 6-month dispensing model reported. Dispensing intervals in Zambia reflected the transition underway at the time of the survey between a standard of care of 3 months per pickup to 6 month dispensing, which is now national policy for stable patients.

### Providers

The final characteristic that helps to describe DSD models is the cadre of staff that provides services. Task-shifting from more to less senior clinical staff, and from clinical staff to lay providers, has been common for many years. Some of the DSD models described by survey respondents developed this practice further, relying more heavily on nonclinical providers located outside facilities, while others continued to make use of different cadres of clinical providers. Table 7 describes the cadres providing clinical care and ARV dispensing in each category of model.

**TABLE 7.** Different Cadres of Clinical Care Providers and Antiretroviral Therapy Dispensers Employed in Differentiated Service Delivery Models for HIV Treatment in Malawi, South Africa, and Zambia

Provider	Number of Organizational-Models				
	Facility-based Individual	Out-of-facility-based Individual	Health Care Worker-led Group	Client-led Group	Total
**Clinical care provider**					
Medical doctor/medical officer/clinical officer	9	1	2	2	14
Nurse	7	28	16	0	51
Community health worker	0	1	0	1	2
Non-specified clinician	3	7	3	2	15
Unclear/not reported	13	6	5	4	28
**Antiretroviral therapy dispenser**					
Pharmacist/pharmacy assistant	12	7	2	0	21
Nurse/clinician	7	7	3	0	17
Community health worker	0	6	5	0	11
Designated patient	0	2	1	9	12
Lay counselor/trained non-clinical personnel	0	14	9	0	23
Medication locker/remote pharmacist	0	3	0	0	3
Unclear/not reported	13	4	6	0	23

In general, individual models relied more on clinical staff (doctors, nurses, and pharmacists), while group models made greater use of lay personnel (community health workers and counselors). The cadre providing these services, though, was frequently not reported, particularly for facility-based individual models.

## DISCUSSION

This survey of organizations implementing differentiated service delivery models for HIV treatment revealed 110 instances of DSD model provision in Malawi, South Africa, and Zambia in 2019. Three of the 4 commonly seen categories of DSD models—facility-based individual care, out-of-facility-based individual care, and health care worker-led groups—were well-represented in all 3 countries; client-led groups were common only in Zambia. Most models continued to provide clinical care at facilities and, as anticipated, most models were limited to stable adult patients. The models described in our survey fell fairly naturally into 12 strategies for service delivery which reflect the evolution and divergence of DSD models since they first emerged. As a set, the 12 strategies may offer a more specific and pragmatic way to describe DSD models for HIV treatment going forward.

Although DSD models are often assumed to be “less-intensive” approaches to service delivery, the models being implemented in 2019 still required relatively frequent interaction between patients and providers. Four interactions per year was most common for stable patients; models that allowed or focused on nonstable patients generally required more interactions. Dispensing intervals also varied by model and country. Models in Zambia and Malawi were beginning to reflect these countries' adoption of 6-month dispensing policies, while dispensing intervals remained relatively short (2–3 months) in South Africa. The advent of COVID-19 and its accompanying restrictions on travel and service delivery may also influence future decisions about how many interactions patients should have with the health care system. Over the course of 2020, models that minimized contact, such as multimonth dispensing and external medication pickup points, have expanded in many countries, while those that were designed to create contact, such as adherence groups, have diminished.^16^^,^^17^

DSD models are also assumed to incorporate task-shifting from more- to less-trained cadres of service providers. In most of the models reported by survey respondents, though, formally trained clinical staff (doctors, nurses, pharmacists) continued to provide the majority of services, even in out-of-facility-based models.

One of the conclusions that can be drawn from our survey is the extent to which the details of each model's design both vary from one another and matter to how well a model achieves the various goals of differentiated service delivery. Within the 4 major categories of DSD models, individual models varied widely, particularly in terms of specific populations targeted and locations and timing of medication pickup or delivery. Home delivery of ARV medications and electronic medication lockers located just outside a clinic facility can both be described as out-of-facility-based individual care, for example, but they differ sharply from one another in terms of resource needs, and they thus potentially have very different roles to play in a national HIV treatment program. There was important variation even within our 12 strategy groups, illustrating the overall difficulty of drawing generalizations about DSD models.

From our survey, we can observe the extent to which the details of each model's design vary from one to another and how the details matter to how well a model achieves the various goals of DSD.

### Limitations

Although we attempted to generate a comprehensive description of DSD models in use in 2019, our survey had many limitations. First, it is possible that our survey missed some implementing organizations in our target countries, and it is unlikely that the inventory of models in Table 1 is truly comprehensive. It includes all the DSD models mentioned by the partners interviewed, but there are almost certainly other approaches being tried by others. We are confident that models that are missing from Table 1, however, are relatively small and/or new initiatives at the time of the survey.

A more important limitation is that the information collected pertains to the situation in 2019, when the survey was conducted. The world of differentiated service delivery is evolving rapidly. Some of the models described to us in 2019 almost certainly no longer exist 2 years later; new models that had not yet been launched in 2019 may be underway now. Similarly, government guidelines for the models being rolled out nationally in each country may also have been updated since the survey was conducted.

Finally, we were not able to weight the models described to us by their importance within national DSD landscapes. As previously mentioned, we attempted to obtain estimates of numbers of facilities and patients participating in each model, but results were incomplete and difficult to interpret. Thus, we could not reliably distinguish between a bespoke, respondent-specific model serving just a handful of patients and a well-established, national model serving tens of thousands. Our data captured the range of diversity but not its scale. Many countries, including the 3 we focused on, are in the process of revising their paper and electronic medical record systems to better capture patient participation in DSD models, but this process had not been completed at the time of our survey. Capturing patient interactions with nonconventional models of care is essential for understanding the extent and impact of DSD models and for managing individual patients' and facilities' performance. (A good source for further information about monitoring and evaluating DSD models can be found at https://cquin.icap.columbia.edu/network-focus-areas/monitoring-and-evaluation-of-dsd/.)

## CONCLUSION

The survey reported here provides what we believe is the most complete description available yet of DSD models for HIV treatment in sub-Saharan Africa. It can both provide examples to other countries of new approaches they have not yet considered and serve as a baseline of model diversity, against which to evaluate the further development of differentiated service delivery in the coming years. For policy makers, understanding the breadth of DSD models being implemented in their own countries is important for learning from local experience. The diversity we observed in such characteristics as the number of health systems interactions required for each model per year, or in the eligibility criteria for different models, is crucial to making decisions about how to optimize the distribution of models across facilities and regions and plan for budget and resource allocation accordingly. Equally important, perhaps, is the recognition of the difficulty of using routinely collected data even to describe DSD models in use, let alone to assess critical outcomes such as coverage and retention in care. Research that overcomes routine data limitations to describe the performance of DSD models—in terms of patient coverage, health outcomes, costs, clinic efficiency, and other consequences—will be of value going forward.

## Supplementary Material

20-00532-Rosen-Supplement1.pdf

20-00532-Rosen-Supplement2.pdf

## Notes

**Peer Reviewed**

**First published online:** May 10, 2021.
