# Supplementary material for: Differentiated Service Delivery Models for HIV Treatment in Malawi, South Africa, and Zambia: A Landscape Analysis
Source: Glob Health Sci Pract. 2021 Jun 30;9(2):296–307. doi: 10.9745/GHSP-D-20-00532 (PMC8324204; doi:10.9745/GHSP-D-20-00532)
Supplement: 20-00532-Rosen-Supplement1.pdf [file 20-00532-Rosen-Supplement1.pdf]

AMBIT Stakeholder Interview

REDCap ID ______________________________

1. Instrument ID ______________________________
(Structure should be: AMBIT20XX)

2. Interviewer(s) name(s) ______________________________

3. Interview date ______________________________
(DD/MM/YYYY)

4. Country
- ☐ Malawi
- ☐ South Africa
- ☐ Zambia

5. Communication mode
- ◯ In-person at respondent's offices
- ◯ In-person at HE2RO
- ◯ In-person other
- ◯ Zoom Skype or Telephone

6. Time start ______________________________

7. Respondent organization name ______________________________

8. Respondent organization website ______________________________

9. Respondent organization telephone number ______________________________

10. Respondent organization address/location ______________________________

"Thank you for agreeing to participate in this interview. My name is ______ and I am a researcher on the AMBIT project team and I will be interviewing you.

AMBIT stands for ALTERNATIVE MODELS OF ART DELIVERY: OPTIMIZING THE BENEFITS. It is a set of data synthesis, data collection, data analysis, and modeling activities aimed at generating information that can be used by ministries, funders, and other policy makers for near- and long-term decision making. It will also help develop an approach and platform for ongoing evaluation of these alternative models of HIV treatment delivery in the future. Primary data analysis is being conducted in three focus countries in sub-Saharan Africa: Malawi, Zambia, and South Africa. AMBIT is funded by the Bill and Melinda Gates Foundation and is scheduled to run until 2022.

We have contacted you because you are knowledgeable about your organization's involvement in differentiated models of service delivery (DSD) for HIV treatment. As part of the AMBIT project, we are estimating coverage (provision and uptake) of DSD in [country], with descriptions of the models in use, which patient groups are targeted for each of these models, and what data are collected to monitor and/or evaluate these programs. From you we would like to learn more about your organization's involvement in the implementation and support of these models.

Please note that we are not conducting human subjects research at this time. We will not record your identity and will not ask you personal questions or ask for your opinions. We only want to know what your organization is doing in the area of differentiated models of care. Please feel free to decline to answer any question that is asked, or to terminate the interview at any time. We will use your organization's name as we compile a report on DSD coverage in [country]. You will have a chance to review the information we include about your organization before the report is made public."

12. Respondent(s) Position (title) ________________________________

13. Respondent(s) role in organization's DSD activities (describe) ____

14. Respondent(s) number of years involved in HIV treatment delivery activities ________________________________

RECORDING

To ensure that we do not miss anything you tell us or record anything in accurately, we would like to make an audio recording of this interview. This will only be for purposes of data accuracy, and the recording will be destroyed as soon as we have completed your organization's entry in our data base. We will not ask or record your name or any personal information. If you are not comfortable with having this interview recorded, we will not do so. We can also stop recording at any time during the interview or delete the recording at the end of the interview if you do not wish us to use it to check our information.

Do you agree that we can record this interview?
◯ Yes
◯ No

Is your organization currently or was your organization previously involved with any projects involving differentiated models of HIV treatment? These may also have been described as decentralization or task-shifting, or decanting strategies for those on ART. We are interested in any ART delivery strategies that your organization is involved in, including support for what you regard as standard care.

First allow provider to respond. Then inquire about some of the following:
Malawi
- Fast Track Refill (FTR)/ Facility Fast Track (FFF)
- Community Adherence Groups (CAG)
- Multi-Month Scripting (MMS)
- Six-Monthly Appointment Program

Zambia
- Community Adherence Groups (CAGs)
- Urban Adherence Groups (UAGs)
- Fast Track (FT)
- Streamlined ART Initiation (START)
- Mobile ART Delivery Program
- Central Chronic Medicine Dispensing and Distribution (CCMDD)

South Africa
- Fast Track Initiation Counselling (FTIC)
- Enhanced Adherence Counselling (EAC)
- Adherence Club (AC)
- Decentralised Medication Delivery (DMD)
- Central Chronic Medicine Dispensing and Distribution (CCMDD)
- Spaced and Fast Lane Appointment Systems (SFLA)
- Tracing and Retention in Care (TRIC)
- Child and Adolescent Disclosure Counselling (CADC)

○ Yes
○ No

Please specify the model or models that are involved in the projects:

☐ Fast Track Refill (FTR)/ Facility Fast Track (FFF)
☐ Community Adherence Groups (CAG)
☐ Multi-Month Scripting (MMS)
☐ Six-Monthly Appointment Program
☐ Community Adherence Groups (CAGs)
☐ Urban Adherence Groups (UAGs)
☐ Fast Track (FT)
☐ Streamlined ART Initiation (START)
☐ Mobile ART Delivery Program
☐ Central Chronic Medicine Dispensing and Distribution (CCMDD)
☐ Fast Track Initiation Counselling (FTIC)
☐ Enhanced Adherence Counselling (EAC)
☐ Adherence Club (AC)
☐ Decentralised Medication Delivery (DMD)
☐ Spaced and Fast Lane Appointment Systems (SFLA)
☐ Tracing and Retention in Care (TRIC)
☐ Child and Adolescent Disclosure Counselling (CADC)
☐ Other

** If other, please specify:

____

For each delivery model mentioned (including standard care), please describe the following characteristics.
Interviewer: start with a detailed narrative description of the model, then fill in the table below. There should be one table for each differentiated model supported by this organization.

SECTION 1: TITLE

Q1. What is the title of the project?

____

Q2. What other organizations do you collaborate with on this project, if any?

____

Q3. How is this project funded?

____

Q4. When did the project start, or when it is scheduled to start?

____
(If day is not known, enter 01 for the day.)

Q5. When is the project expected to end, if ending?

- ◯ Project has an end date
- ◯ No end date for the project

Q5a. ** When is it expected to end?

____
(If day is not known, enter 01 for the day.)

Q6. What is your organization's role in this project?

____
(Direct service delivery (provide staff, infrastructure, etc.), routine monitoring and evaluation, research, training)

SECTION 2: MODEL OF CARE

Q7. What is the model of care called?

____
(Include both the organization's label for the model and, if possible, a standard label from the list above.)

Q8. Please describe the model in detail.

____
(Record and take notes on description.)

Q9. What is the model primarily trying to achieve?

- ☐ Improved adherence
- ☐ Improved retention
- ☐ VL suppression
- ☐ Decongestion of clinics
- ☐ Cost saving
- ☐ Ensuring confidentiality (e.g. for key pop. models)
- ☐ Improved psycho-social support
- ☐ Other

Q9a. ** If other, please specify

____

SECTION 3: POPULATION ELIGIBLE
Who is the population group eligible for this model?

Q10. HIV treatment status

- ◯ Stable
- ◯ Not stable/ detectable viral load
- ◯ Patients with comorbidities
- ◯ Newly initiated
- ◯ Other

Q10a. ** If other, please specify

____

Q10b. ** If model is for stable patients, how is "stable" defined? (Criteria for eligibility)

____

Q10c. ** If model is for unstable patients, how is "unstable" defined (criteria for eligibility)

____

Q11. Ages eligible

- ☐ No age eligibility criteria
- ☐ Adults (>18)
- ☐ Adolescents/Youth
- ☐ Children
- ☐ Neonates
- ☐ Other

Q11a. ** If adolescents/youth, please specify age range.

____

Q11b. ** If children, please specify age range.

____

Q11c. ** If other, please specify

____

Q12. Are there any health conditions included in model in addition to ART?

- ☐ None
- ☐ TB
- ☐ Non-communicable diseases (hypertension, diabetes)
- ☐ STDs/STIs
- ☐ Reproductive health conditions
- ☐ Antenatal care/ANC/PNC
- ☐ Mental health conditions
- ☐ Other

Q12a. ** If other, please specify

____

Q13. Regions/districts included (list names)

____

Q14. Other population characteristics	☐ No other population characteristics<br>☐ Women only<br>☐ Men only<br>☐ Pregnant women only<br>☐ Key populations (MSM, FSW, PWID, young women, adolescents)<br>☐ Other
Q14a. ** If other, please specify	__________________________________
Q14b. ** If a key population, specify the population	☐ Men who have sex with men (MSM)<br>☐ Female sex workers (FSW)<br>☐ People who inject drugs (PWID)<br>☐ Young women<br>☐ Transgender<br>☐ Children<br>☐ Adolescents<br>☐ Infants<br>☐ Other
Q14c. ** If other key population, please specify	__________________________________
SECTION 4: INTENSITY (WHAT)	
Q15. What specific services are provided?	☐ Clinical consultation<br>☐ Drug delivery<br>☐ Drug dispensation/refill<br>☐ VL testing<br>☐ Adherence/retention counseling<br>☐ Mental health counseling<br>☐ EID diagnosis of HIV<br>☐ TB/RIF testing and care<br>☐ STI/STD testing care<br>☐ PMTCT<br>☐ Food assistance<br>☐ Health education<br>☐ Up-referral<br>☐ HIV testing (this may not be relevant)<br>☐ Other
Q15a. ** If other, please specify	__________________________________
Q16. Please note whether any of the above services are integrated with service delivery:	____
SECTION 5: LOCATION (WHERE)<br>Where are the following ART and related services delivered?	
Q17. HIV testing	__________________________________<br>(E.g. Facility (specify level), community (specify where), medication pick up point (specify where), household)

Q18. ART initiation

____
(E.g. Facility (specify level), community (specify where), medication pick up point (specify where), household)

Q19. ART medication refills

____
(E.g. Facility (specify level), community (specify where), medication pick up point (specify where), household)

Q20. Medical monitoring visits

____
(E.g. Facility (specify level), community (specify where), medication pick up point (specify where), household)

Q21. Blood draw for HIV viral load tests

____
(E.g. Facility (specify level), community (specify where), medication pick up point (specify where), household)

Q22. Blood draw for HIV CD4 counts (if done)

____
(E.g. Facility (specify level), community (specify where), medication pick up point (specify where), household)

SECTION 6: FREQUENCY (WHEN)
How often are services provided?

Q23. Frequency of interactions with providers.

____
(Specify which type of provider, how many times per year)

Q24. Minimum number of times patient must come to fixed clinic building per year

____

Q25. How many months supply is given per ART dispensation?

____
(Note the number of months)

SECTION 7: PROVIDER CADRE(S) (WHO)
Who is providing services?

Q26. Service delivery sector

____
(E.g. Public sector through the government managed public health infrastructure, Partner/NGO programs or services which service the public sector)

Q27. Provider(s) involved

(e.g. Clinician/doctor, nurse, CHW, expert patient, pharmacy staff, lay counselor, lab technician)

____
(Specify cadre(s) of healthcare workers who deliver services and which services are assigned to each cadre)

SECTION 8: OTHER CHARACTERISTICS

Q28. Are there are additional components to the program, either for HIV or non-HIV patients, that have not been mentioned yet?

____

SECTION 9: SCALE

Q29. How many patients are enrolled in this model of care?

____

Q30. How does your project define "enrolled"- ever enrolled, utilized at most recent interaction, consistently utilized?

____

Q31. How many patients did the project report in your most recent report?

____
(Specify patient type if known (e.g. adherent, retained, ever enrolled))

SECTION 10: DATA AND DOCUMENTATION

Q32. Are patient volumes reported by sites verified?

◯ Yes
◯ No

Q33. What kind of information do you collect as you implement the intervention? What measures do you track and how?

____

Q34. Are the patients enrolled in the model registered in an electronic medical record system (Baobab, TIER.Net, Smartcare)?

If yes, please specify the EMRS system.

____

Q35. If yes, does the EMRS capture their participation in this model of care? Specifically, what fields are used to indicate model participation?

____

Q36. If paper patient records are used, do they capture patient participation in the model? Enrollment only, or at every visit?

____

Q37. Do you manage a specific database with patient-level data for this project, separate from the national EMRS? If so, what fields are in it and is it possible that we could access it (anonymized and with appropriate ethics approval)?

____

Q38. Do you have facility- or community-level aggregate data that you could share with us (e.g. facility survey)?

____
(Probe- Where and how is this recorded?)

SECTION 11: EXISTING EVALUATIONS

Q39. What aspects of the project have you or will you evaluate?

- ☐ Coverage
- ☐ Uptake
- ☐ Outcome
- ☐ Cost or resource allocation
- ☐ Acceptability
- ☐ Feasibility
- ☐ Other

Q39a. ** If other, please specify

____

Q40. How are the outcomes measured?

____

Q41. What data are collected?

____

Q42. What is the frequency of data collection?

____

Q43. How long is the follow up time for outcomes?

____

SECTION 12: DOCUMENTATION

Q44. Are there any publications, protocols, guidelines, reports, toolkits, M&E plans, presentations, or other documents about this project that you can share with us?

____
(For each document, ask about whether we can share it outside the AMBIT team)

Q45. Have any mapping exercises been done to illustrate facility-level implementation of DSD and/or patient uptake or coverage? Can we access the results?

____

Q46. Could you share the codebook or data dictionary for any data sets you collect?

____

Q47. Are there blank copies of questionnaires, survey instruments, clinical stationery (file templates), or other data collection instruments you use that could be shared with us?

____

Q48. Are there others in or collaborating with your organization we should interview about other DSD projects or related topics?	________________________
Q49. Do you have any cost estimates for the model, either from a costing exercise or your own expenditure records?<br><br>If yes, please specify the type of cost estimates that you have.	________________________
SECTION 13: RESEARCH PROJECTS	
Q50. Is this project a research or evaluation study?	◯ Yes<br>◯ No
Q51. What is the design of the study or evaluation you are conducting?	◯ Individual-level RCT<br>◯ Cluster RCT<br>◯ Prospective evaluation<br>◯ Retrospective evaluation<br>◯ Cross-sectional<br>◯ Mixed-methods<br>◯ Qualitative<br>◯ Other
Q51a. ** If other, please specify	____________________
Q52. Who is included in the study population?	________________________<br>(E.g. all patients enrolled in model, all patients managed by facility, etc.)
Q53. Is there a comparison group?	◯ Yes<br>◯ No
Q53a. ** If yes, please add details about the comparison group:	________________________<br>(E.g. Control facilities, Pre-post design)
Q54. What time period will your data represent?	____________________<br>(Specify start date and end date if known.)
SECTION 14: ADDITIONAL INFORMATION	
Q55. Is this model recommended in national guidelines for ART delivery? Is it supported by any other implementing partner/organization? If you left would this effort completely stop?	________________________
Q56. Is the Department or Ministry of Health a partner in implementing this model?	◯ Yes<br>◯ No

Q56a. ** If yes, please specify the DoH or MoH's involvement:	__________________________________
Q57. Please make any additional comments on the intervention here. Is there anything about the model or your organization's involvement that we have not addressed yet?	__________________________________
END SPECIFIC MODEL CHARACTERISTICS	
Are there additional projects the partner would like to report on?	◯ Yes<br>◯ No
PROJECT 2: SECTION 1: TITLE	
P2 Q1. What is the title of the project?	__________________________________
P2 Q2. What other organizations do you collaborate with on this project, if any?	__________________________________
P2 Q3. How is this project funded?	__________________________________
P2 Q4. When did the project start, or when it is scheduled to start?	__________________________________<br>(If day is not known, enter 01 for the day.)
P2 Q5. When is the project expected to end, if ending?	◯ Project has an end date<br>◯ No end date for the project
P2 Q5a. ** When is it expected to end?	__________________________________<br>(If day is not known, enter 01 for the day.)
P2 Q6. What is your organization's role in this project?	__________________________________<br>(Direct service delivery (provide staff, infrastructure, etc.), routine monitoring and evaluation, research, training)
PROJECT 2: SECTION 2: MODEL OF CARE	
P2 Q7. What is the model of care called?	__________________________________<br>(Include both the organization's label for the model and, if possible, a standard label from the list above.)
P2 Q8. Please describe the model in detail.	__________________________________<br>(Record and take notes on description.)

P2 Q9. What is the model primarily trying to achieve?	☐ Improved adherence<br>☐ Improved retention<br>☐ VL suppression<br>☐ Decongestion of clinics<br>☐ Cost saving<br>☐ Ensuring confidentiality (e.g. for key pop. models)<br>☐ Improved psycho-social support<br>☐ Other
P2 Q9a. ** If other, please specify	__________________________________
PROJECT 2: SECTION 3: POPULATION ELIGIBLE<br>Who is the population group eligible for this model?	
P2 Q10. HIV treatment status	◯ Stable<br>◯ Not stable/ detectable viral load<br>◯ Patients with comorbidities<br>◯ Newly initiated<br>◯ Other
P2 Q10a. ** If other, please specify	__________________________________
P2 Q10b. ** If model is for stable patients, how is "stable" defined? (Criteria for eligibility)	____
P2 Q10c. ** If model is for unstable patients, how is "unstable" defined (criteria for eligibility)	__________________________________
P2 Q11. Ages eligible	☐ No age eligibility criteria<br>☐ Adults (>18)<br>☐ Adolescents/Youth<br>☐ Children<br>☐ Neonates<br>☐ Other
P2 Q11a. ** If adolescents/youth, please specify age range.	__________________________________
P2 Q11b. ** If children, please specify age range.	__________________________________
P2 Q11c. ** If other, please specify	__________________________________
P2 Q12. Are there any health conditions included in model in addition to ART?	☐ None<br>☐ TB<br>☐ Non-communicable diseases (hypertension, diabetes)<br>☐ STDs/STIs<br>☐ Reproductive health conditions<br>☐ Antenatal care/ANC/PNC<br>☐ Mental health conditions<br>☐ Other

P2 Q12a. ** If other, please specify

____

P2 Q13. Regions/districts included (list names)

____

P2 Q14. Other population characteristics

- ☐ No other population characteristics
- ☐ Women only
- ☐ Men only
- ☐ Pregnant women only
- ☐ Key populations (MSM, FSW, PWID, young women, adolescents)
- ☐ Other

P2 Q14a. ** If other, please specify

____

P2 Q14b. ** If a key population, specify the population

- ☐ Men who have sex with men (MSM)
- ☐ Female sex workers (FSW)
- ☐ People who inject drugs (PWID)
- ☐ Young women
- ☐ Transgender
- ☐ Children
- ☐ Adolescents
- ☐ Infants
- ☐ Other

P2 Q14c. ** If other key population, please specify

____

PROJECT 2: SECTION 4: INTENSITY (WHAT)

P2 Q15. What specific services are provided?

- ☐ Clinical consultation
- ☐ Drug delivery
- ☐ Drug dispensation/refill
- ☐ VL testing
- ☐ Adherence/retention counseling
- ☐ Mental health counseling
- ☐ EID diagnosis of HIV
- ☐ TB/RIF testing and care
- ☐ STI/STD testing care
- ☐ PMTCT
- ☐ Food assistance
- ☐ Health education
- ☐ Up-referral
- ☐ HIV testing (this may not be relevant)
- ☐ Other

P2 Q15a. ** If other, please specify

____

P2 Q16. Please note whether any of the above services are integrated with service delivery:

____

PROJECT 2: SECTION 5: LOCATION (WHERE)
Where are the following ART and related services delivered?

P2 Q17. HIV testing

____
(E.g. Facility (specify level), community (specify where), medication pick up point (specify where), household)

P2 Q18. ART initiation

____
(E.g. Facility (specify level), community (specify where), medication pick up point (specify where), household)

P2 Q19. ART medication refills

____
(E.g. Facility (specify level), community (specify where), medication pick up point (specify where), household)

P2 Q20. Medical monitoring visits

____
(E.g. Facility (specify level), community (specify where), medication pick up point (specify where), household)

P2 Q21. Blood draw for HIV viral load tests

____
(E.g. Facility (specify level), community (specify where), medication pick up point (specify where), household)

P2 Q22. Blood draw for HIV CD4 counts (if done)

____
(E.g. Facility (specify level), community (specify where), medication pick up point (specify where), household)

PROJECT 2: SECTION 6: FREQUENCY (WHEN)
How often are services provided?

P2 Q23. Frequency of interactions with providers.

____
(Specify which type of provider, how many times per year)

P2 Q24. Minimum number of times patient must come to fixed clinic building per year

____

P2 Q25. How many months supply is given per ART dispensation?

____
(Note the number of months)

PROJECT 2: SECTION 7: PROVIDER CADRE(S) (WHO)
Who is providing services?

P2 Q26. Service delivery sector

____
(E.g. Public sector through the government managed public health infrastructure, Partner/NGO programs or services which service the public sector)

P2 Q27. Provider(s) involved

(e.g. Clinician/doctor, nurse, CHW, expert patient, pharmacy staff, lay counselor, lab technician)

____
(Specify cadre(s) of healthcare workers who deliver services and which services are assigned to each cadre)

PROJECT 2: SECTION 8: OTHER CHARACTERISTICS

P2 Q28. Are there are additional components to the program, either for HIV or non-HIV patients, that have not been mentioned yet?

____

PROJECT 2: SECTION 9: SCALE

P2 Q29. How many patients are enrolled in this model of care?

____

P2 Q30. How does your project define "enrolled"- ever enrolled, utilized at most recent interaction, consistently utilized?

____

P2 Q31. How many patients did the project report in your most recent report?

____
(Specify patient type if known (e.g. adherent, retained, ever enrolled))

PROJECT 2: SECTION 10: DATA AND DOCUMENTATION

P2 Q32. Are patient volumes reported by sites verified?

◯ Yes
◯ No

P2 Q33. What kind of information do you collect as you implement the intervention? What measures do you track and how?

____

P2 Q34. Are the patients enrolled in the model registered in an electronic medical record system (Baobab, TIER.Net, Smartcare)?

If yes, please specify the EMRS system.

____

P2 Q35. If yes, does the EMRS capture their participation in this model of care? Specifically, what fields are used to indicate model participation?

____

P2 Q36. If paper patient records are used, do they capture patient participation in the model? Enrollment only, or at every visit?

____

P2 Q37. Do you manage a specific database with patient-level data for this project, separate from the national EMRS? If so, what fields are in it and is it possible that we could access it (anonymized and with appropriate ethics approval)?	__________________________________
P2 Q38. Do you have facility- or community-level aggregate data that you could share with us (e.g. facility survey)?	__________________________________<br>(Probe- Where and how is this recorded?)
PROJECT 2: SECTION 11: EXISTING EVALUATIONS	
P2 Q39. What aspects of the project have you or will you evaluate?	☐ Coverage<br>☐ Uptake<br>☐ Outcome<br>☐ Cost or resource allocation<br>☐ Acceptability<br>☐ Feasibility<br>☐ Other
P2 Q39a. ** If other, please specify	____________________________
P2 Q40. How are the outcomes measured?	__________________________________
P2 Q41. What data are collected?	__________________________________
P2 Q42. What is the frequency of data collection?	__________________________________
P2 Q43. How long is the follow up time for outcomes?	____________________________
PROJECT 2: SECTION 12: DOCUMENTATION	
P2 Q44. Are there any publications, protocols, guidelines, reports, toolkits, M&E plans, presentations, or other documents about this project that you can share with us?	__________________________________<br>(For each document, ask about whether we can share it outside the AMBIT team)
P2 Q45. Have any mapping exercises been done to illustrate facility-level implementation of DSD and/or patient uptake or coverage? Can we access the results?	__________________________________
P2 Q46. Could you share the codebook or data dictionary for any data sets you collect?	__________________________________

P2 Q47. Are there blank copies of questionnaires, survey instruments, clinical stationery (file templates), or other data collection instruments you use that could be shared with us?	__________________________________
P2 Q48. Are there others in or collaborating with your organization we should interview about other DSD projects or related topics?	__________________________________
P2 Q49. Do you have any cost estimates for the model, either from a costing exercise or your own expenditure records?<br><br>If yes, please specify the type of cost estimates that you have.	__________________________________
PROJECT 2: SECTION 13: RESEARCH PROJECTS	
P2 Q50. Is this project a research or evaluation study?	◯ Yes<br>◯ No
P2 Q51. What is the design of the study or evaluation you are conducting?	◯ Individual-level RCT<br>◯ Cluster RCT<br>◯ Prospective evaluation<br>◯ Retrospective evaluation<br>◯ Cross-sectional<br>◯ Mixed-methods<br>◯ Qualitative<br>◯ Other
P2 Q51a. ** If other, please specify	___________________________
P2 Q52. Who is included in the study population?	__________________________________<br>(E.g. all patients enrolled in model, all patients managed by facility, etc.)
P2 Q53. Is there a comparison group?	◯ Yes<br>◯ No
P2 Q53a. ** If yes, please add details about the comparison group:	__________________________________<br>(E.g. Control facilities, Pre-post design)
P2 Q54. What time period will your data represent?	___________________________<br>(Specify start date and end date if known.)
PROJECT 2: SECTION 14: ADDITIONAL INFORMATION	
P2 Q55. Is this model recommended in national guidelines for ART delivery? Is it supported by any other implementing partner/organization? If you left would this effort completely stop?	__________________________________

P2 Q56. Is the Department or Ministry of Health a partner in implementing this model?	◯ Yes<br>◯ No
P2 Q56a. ** If yes, please specify the DoH or MoH's involvement:	__________________________________
P2 Q57. Please make any additional comments on the intervention here. Is there anything about the model or your organization's involvement that we have not addressed yet?	__________________________________
END SPECIFIC MODEL CHARACTERISTICS	
Are there additional projects the partner would like to report on?	◯ Yes<br>◯ No
PROJECT 3: SECTION 1: TITLE	
P3 Q1. What is the title of the project?	__________________________
P3 Q2. What other organizations do you collaborate with on this project, if any?	__________________________
P3 Q3. How is this project funded?	__________________________________
P3 Q4. When did the project start, or when it is scheduled to start?	__________________________<br>(If day is not known, enter 01 for the day.)
P3 Q5. When is the project expected to end, if ending?	◯ Project has an end date<br>◯ No end date for the project
P3 Q5a. ** When is it expected to end?	__________________________<br>(If day is not known, enter 01 for the day.)
P3 Q6. What is your organization's role in this project?	__________________________________<br>(Direct service delivery (provide staff, infrastructure, etc.), routine monitoring and evaluation, research, training)
PROJECT 3: SECTION 2: MODEL OF CARE	
P3 Q7. What is the model of care called?	__________________________<br>(Include both the organization's label for the model and, if possible, a standard label from the list above.)

P3 Q8. Please describe the model in detail.

____
(Record and take notes on description.)

P3 Q9. What is the model primarily trying to achieve?

- ☐ Improved adherence
- ☐ Improved retention
- ☐ VL suppression
- ☐ Decongestion of clinics
- ☐ Cost saving
- ☐ Ensuring confidentiality (e.g. for key pop. models)
- ☐ Improved psycho-social support
- ☐ Other

P3 Q9a. ** If other, please specify

____

PROJECT 3: SECTION 3: POPULATION ELIGIBLE
Who is the population group eligible for this model?

P3 Q10. HIV treatment status

- ◯ Stable
- ◯ Not stable/ detectable viral load
- ◯ Patients with comorbidities
- ◯ Newly initiated
- ◯ Other

P3 Q10a. ** If other, please specify

____

P3 Q10b. ** If model is for stable patients, how is "stable" defined? (Criteria for eligibility)

____

P3 Q10c. ** If model is for unstable patients, how is "unstable" defined (criteria for eligibility)

____

P3 Q11. Ages eligible

- ☐ No age eligibility criteria
- ☐ Adults (>18)
- ☐ Adolescents/Youth
- ☐ Children
- ☐ Neonates
- ☐ Other

P3 Q11a. ** If adolescents/youth, please specify age range.

____

P3 Q11b. ** If children, please specify age range.

____

P3 Q11c. ** If other, please specify

____

P3 Q12. Are there any health conditions included in model in addition to ART?	☐ None<br>☐ TB<br>☐ Non-communicable diseases (hypertension, diabetes)<br>☐ STDs/STIs<br>☐ Reproductive health conditions<br>☐ Antenatal care/ANC/PNC<br>☐ Mental health conditions<br>☐ Other
P3 Q12a. ** If other, please specify	__________________________________
P3 Q13. Regions/districts included (list names)	____
P3 Q14. Other population characteristics	☐ No other population characteristics<br>☐ Women only<br>☐ Men only<br>☐ Pregnant women only<br>☐ Key populations (MSM, FSW, PWID, young women, adolescents)<br>☐ Other
P3 Q14a. ** If other, please specify	__________________________________
P3 Q14b. ** If a key population, specify the population	☐ Men who have sex with men (MSM)<br>☐ Female sex workers (FSW)<br>☐ People who inject drugs (PWID)<br>☐ Young women<br>☐ Transgender<br>☐ Children<br>☐ Adolescents<br>☐ Infants<br>☐ Other
P3 Q14c. ** If other key population, please specify	__________________________________
PROJECT 3: SECTION 4: INTENSITY (WHAT)	
P3 Q15. What specific services are provided?	☐ Clinical consultation<br>☐ Drug delivery<br>☐ Drug dispensation/refill<br>☐ VL testing<br>☐ Adherence/retention counseling<br>☐ Mental health counseling<br>☐ EID diagnosis of HIV<br>☐ TB/RIF testing and care<br>☐ STI/STD testing care<br>☐ PMTCT<br>☐ Food assistance<br>☐ Health education<br>☐ Up-referral<br>☐ HIV testing (this may not be relevant)<br>☐ Other
P3 Q15a. ** If other, please specify	__________________________________

P3 Q16. Please note whether any of the above services are integrated with service delivery:

____

PROJECT 3: SECTION 5: LOCATION (WHERE)
Where are the following ART and related services delivered?

P3 Q17. HIV testing

____
(E.g. Facility (specify level), community (specify where), medication pick up point (specify where), household)

P3 Q18. ART initiation

____
(E.g. Facility (specify level), community (specify where), medication pick up point (specify where), household)

P3 Q19. ART medication refills

____
(E.g. Facility (specify level), community (specify where), medication pick up point (specify where), household)

P3 Q20. Medical monitoring visits

____
(E.g. Facility (specify level), community (specify where), medication pick up point (specify where), household)

P3 Q21. Blood draw for HIV viral load tests

____
(E.g. Facility (specify level), community (specify where), medication pick up point (specify where), household)

P3 Q22. Blood draw for HIV CD4 counts (if done)

____
(E.g. Facility (specify level), community (specify where), medication pick up point (specify where), household)

PROJECT 3: SECTION 6: FREQUENCY (WHEN)
How often are services provided?

P3 Q23. Frequency of interactions with providers.

____
(Specify which type of provider, how many times per year)

P3 Q24. Minimum number of times patient must come to fixed clinic building per year

____

P3 Q25. How many months supply is given per ART dispensation?

____
(Note the number of months)

PROJECT 3: SECTION 7: PROVIDER CADRE(S) (WHO)
Who is providing services?

P3 Q26. Service delivery sector

____
(E.g. Public sector through the government managed public health infrastructure, Partner/NGO programs or services which service the public sector)

P3 Q27. Provider(s) involved

(e.g. Clinician/doctor, nurse, CHW, expert patient, pharmacy staff, lay counselor, lab technician)

____
(Specify cadre(s) of healthcare workers who deliver services and which services are assigned to each cadre)

PROJECT 3: SECTION 8: OTHER CHARACTERISTICS

P3 Q28. Are there are additional components to the program, either for HIV or non-HIV patients, that have not been mentioned yet?

____

PROJECT 3: SECTION 9: SCALE

P3 Q29. How many patients are enrolled in this model of care?

____

P3 Q30. How does your project define "enrolled"- ever enrolled, utilized at most recent interaction, consistently utilized?

____

P3 Q31. How many patients did the project report in your most recent report?

____
(Specify patient type if known (e.g. adherent, retained, ever enrolled))

PROJECT 3: SECTION 10: DATA AND DOCUMENTATION

P3 Q32. Are patient volumes reported by sites verified?

- ◯ Yes
- ◯ No

P3 Q33. What kind of information do you collect as you implement the intervention? What measures do you track and how?

____

P3 Q34. Are the patients enrolled in the model registered in an electronic medical record system (Baobab, TIER.Net, Smartcare)?

If yes, please specify the EMRS system.

____

P3 Q35. If yes, does the EMRS capture their participation in this model of care? Specifically, what fields are used to indicate model participation?

____

P3 Q36. If paper patient records are used, do they capture patient participation in the model? Enrollment only, or at every visit?	__________________________________
P3 Q37. Do you manage a specific database with patient-level data for this project, separate from the national EMRS? If so, what fields are in it and is it possible that we could access it (anonymized and with appropriate ethics approval)?	__________________________________
P3 Q38. Do you have facility- or community-level aggregate data that you could share with us (e.g. facility survey)?	__________________________________<br>(Probe- Where and how is this recorded?)
PROJECT 3: SECTION 11: EXISTING EVALUATIONS	
P3 Q39. What aspects of the project have you or will you evaluate?	☐ Coverage<br>☐ Uptake<br>☐ Outcome<br>☐ Cost or resource allocation<br>☐ Acceptability<br>☐ Feasibility<br>☐ Other
P3 Q39a. ** If other, please specify	____________________________
P3 Q40. How are the outcomes measured?	__________________________________
P3 Q41. What data are collected?	__________________________________
P3 Q42. What is the frequency of data collection?	__________________________________
P3 Q43. How long is the follow up time for outcomes?	____________________________
PROJECT 3: SECTION 12: DOCUMENTATION	
P3 Q44. Are there any publications, protocols, guidelines, reports, toolkits, M&E plans, presentations, or other documents about this project that you can share with us?	__________________________________<br>(For each document, ask about whether we can share it outside the AMBIT team)
P3 Q45. Have any mapping exercises been done to illustrate facility-level implementation of DSD and/or patient uptake or coverage? Can we access the results?	__________________________________

P3 Q46. Could you share the codebook or data dictionary for any data sets you collect?	__________________________________
P3 Q47. Are there blank copies of questionnaires, survey instruments, clinical stationery (file templates), or other data collection instruments you use that could be shared with us?	__________________________________
P3 Q48. Are there others in or collaborating with your organization we should interview about other DSD projects or related topics?	__________________________________
P3 Q49. Do you have any cost estimates for the model, either from a costing exercise or your own expenditure records?<br><br>If yes, please specify the type of cost estimates that you have.	__________________________________
PROJECT 3: SECTION 13: RESEARCH PROJECTS	
P3 Q50. Is this project a research or evaluation study?	◯ Yes<br>◯ No
P3 Q51. What is the design of the study or evaluation you are conducting?	◯ Individual-level RCT<br>◯ Cluster RCT<br>◯ Prospective evaluation<br>◯ Retrospective evaluation<br>◯ Cross-sectional<br>◯ Mixed-methods<br>◯ Qualitative<br>◯ Other
P3 Q51a. ** If other, please specify	____________________________
P3 Q52. Who is included in the study population?	__________________________________<br>(E.g. all patients enrolled in model, all patients managed by facility, etc.)
P3 Q53. Is there a comparison group?	◯ Yes<br>◯ No
P3 Q53a. ** If yes, please add details about the comparison group:	__________________________________<br>(E.g. Control facilities, Pre-post design)
P3 Q54. What time period will your data represent?	____________________________<br>(Specify start date and end date if known.)
PROJECT 3: SECTION 14: ADDITIONAL INFORMATION	

P3 Q55. Is this model recommended in national guidelines for ART delivery? Is it supported by any other implementing partner/organization? If you left would this effort completely stop?	__________________________________
P3 Q56. Is the Department or Ministry of Health a partner in implementing this model?	◯ Yes<br>◯ No
P3 Q56a. ** If yes, please specify the DoH or MoH's involvement:	__________________________________
P3 Q57. Please make any additional comments on the intervention here. Is there anything about the model or your organization's involvement that we have not addressed yet?	__________________________________
END SPECIFIC MODEL CHARACTERISTICS	
16. Does the organization have any plans for future projects or programs, beyond those that we have already discussed, that include differentiated models of care? What and where will these be implemented? Can we use this information in describing what to anticipate in DSD development in the future?	__________________________________
17. We have created a list of relevant stakeholders and databases that we plan to use to document the work that is being done on differentiated care. Would you be willing to review our stakeholder and database list for [country] and provide input or information you think we may have missed? If you agree, we will send the list to you shortly by e-mail.	◯ Yes<br>◯ No
17a. Confirm email address:	____________________________
18. Is there anything else you'd like to tell me before we finish?	__________________________________
19. Confirm decision about recording this interview and sharing documents.<br><br>Confirmed?	◯ Yes<br>◯ No
20. Confirm response about the possibility of accessing organization's site- or patient-level data in the future.<br><br>Confirmed?	◯ Yes<br>◯ No
21. Confirm decision about review of stakeholder and data set lists and record respondent's e-mail address.<br><br>Confirmed?	◯ Yes<br>◯ No

22. Can we contact the respondent(s) again if we have further questions or to clarify the information we've received?

◯ Yes
◯ No

THANK RESPONDENT(S) FOR THEIR TIME AND ASSISTANCE.

Record time interview ended ____________________.

____

Please note any additional comments on the interview.

____
