# Supplementary material for: Differentiated Service Delivery Models for HIV Treatment in Malawi, South Africa, and Zambia: A Landscape Analysis
Source: Glob Health Sci Pract. 2021 Jun 30;9(2):296–307. doi: 10.9745/GHSP-D-20-00532 (PMC8324204; doi:10.9745/GHSP-D-20-00532)
Supplement: 20-00532-Rosen-Supplement2.pdf [file 20-00532-Rosen-Supplement2.pdf]

Supplement to: Huber A, Pascoe S, Nichols B, et al. Differentiated service delivery models for HIV treatment in Malawi, South Africa, and Zambia: A landscape analysis. *Glob Health Sci Pract*. 2021;9(2).
https://doi.org/10.9745/GHSP-D-20-00532

Supplement 2. Differentiated Service Delivery Model Domains

Domain	Question asked	Definition
Population	Who does the model serve?	Population is categorized by patient ART status (stable, newly initiated, not stable) and by age (child, adolescent, adult); occasionally also by vulnerability group (general, men who have sex with men (MSM), adolescents etc.).
Location	Is care provided in the clinic or off-site?	Location is categorized as 1) receiving all services at a fixed health care facility, 2) receiving all services outside of health care facility; or 3) mixed, receiving some services in each location.
Frequency	How often does the patient interact with a healthcare provider or facility?	Number of unique times a client receives any service per 12-month period. For example, a client receiving a combined clinical consultation and drug pickup at the same place on the same day every 3 months has a frequency of 4.
Dispensing interval	How many months of medications are dispensed?	Number of months of ARV medications provided at each interaction with the healthcare system. This indicator measures dispensing, not prescribing (e.g. a six-month prescription delivered twice, in three-month quantities each, has a three month duration).
Provider	Which cadres of clinical and/or lay staff provide the services?	Potential cadres include doctors, nurses, lay counselors, community health workers, pharmacists, and others designated within the models.

Adapted from: Duncombe C, Rosenblum S, Hellmann N, et al. Reframing HIV care: putting people at the centre of antiretroviral delivery. *Trop Med Int Health*. 2015;20(4):430-447.
